# Supplementary material for: The causal role of circulating inflammatory markers in osteoporosis: a bidirectional Mendelian randomized study
Source: Front Immunol. 2024 Jul 18;15:1412298. doi: 10.3389/fimmu.2024.1412298 (PMC11291241; doi:10.3389/fimmu.2024.1412298)
Supplement: Supplementary file 1 [file DataSheet_1.zip › supplementary material/CIM-OSTEOPOROSIS /GCST90274835.csv_forest.pdf]

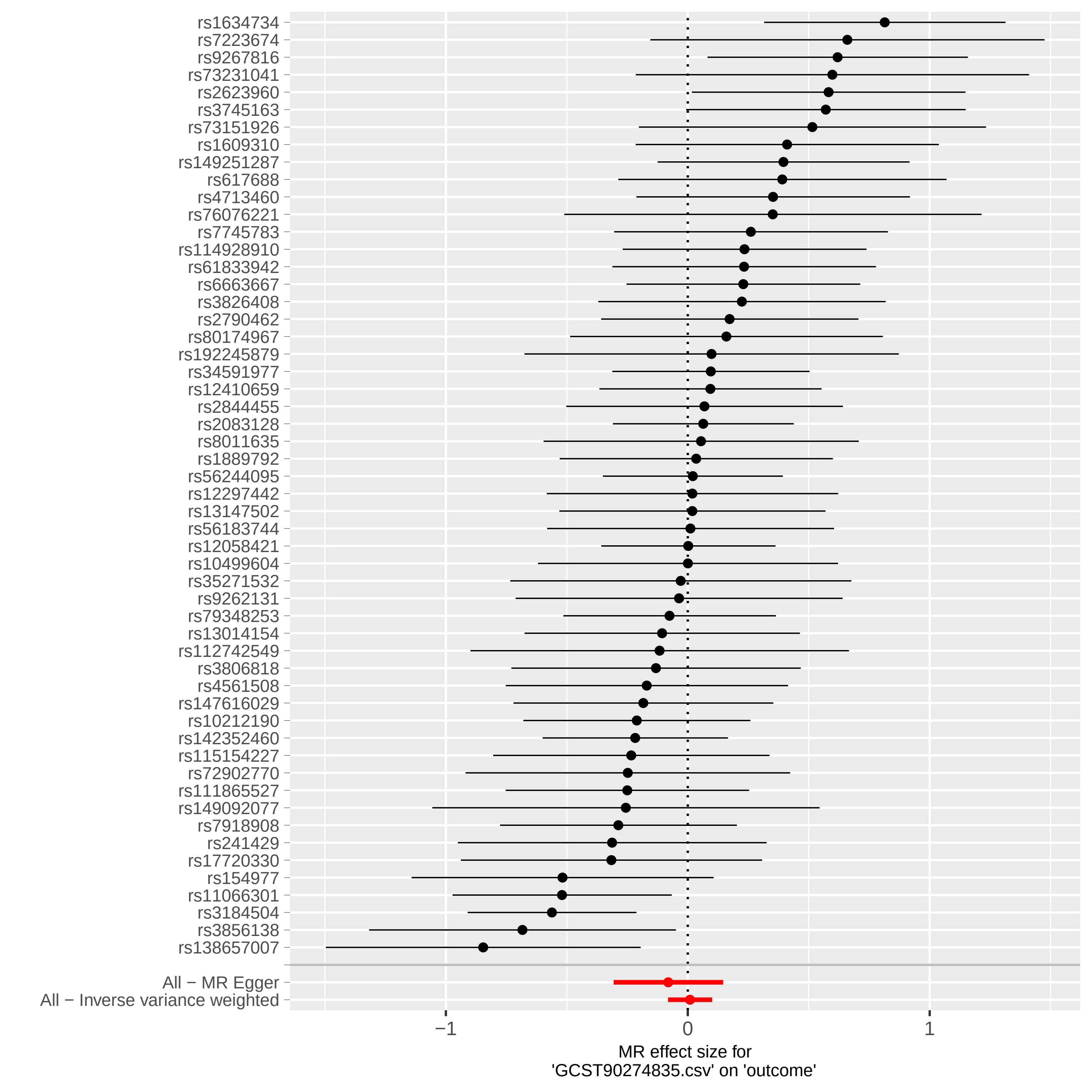
rs1634734
rs7223674
rs9267816
rs73231041
rs2623960
rs3745163
rs73151926
rs1609310
rs149251287
rs617688
rs4713460
rs76076221
rs7745783
rs114928910
rs61833942
rs6663667
rs3826408
rs2790462
rs80174967
rs192245879
rs34591977
rs12410659
rs2844455
rs2083128
rs8011635
rs1889792
rs56244095
rs12297442
rs13147502
rs56183744
rs12058421
rs10499604
rs35271532
rs9262131
rs79348253
rs13014154
rs112742549
rs3806818
rs4561508
rs147616029
rs10212190
rs142352460
rs115154227
rs72902770
rs111865527
rs149092077
rs7918908
rs241429
rs17720330
rs154977
rs11066301
rs3184504
rs3856138
rs138657007
All − MR Egger
All − Inverse variance weighted
−1
0
1
MR effect size for
'GCST90274835.csv' on 'outcome'
